# Tuberculosis and nutrition

**DOI:** 10.4103/0970-2113.45198

**Published:** 2009

**Authors:** Krishna Bihari Gupta, Rajesh Gupta, Atulya Atreja, Manish Verma, Suman Vishvkarma

**Affiliations:** *Department of Tuberculosis and Respiratory Medicine, Pt. Bhagwat Dayal Sharma Post-Graduate Institute of Medical Sciences, Rohtak, India*

**Keywords:** Antituberculosis chemotherapy, human immunodeficiency virus (HIV) infection, malnutrition, micronutrients, tuberculosis

## Abstract

Malnutrition and tuberculosis are both problems of considerable magnitude in most of the underdeveloped regions of the world. These two problems tend to interact with each other. Tuberculosis mortality rates in different economic groups in a community tend to vary inversely with their economic levels. Similarly, nutritional status is significantly lower in patients with active tuberculosis compared with healthy controls. Malnutrition can lead to secondary immunodeficiency that increases the host's susceptibility to infection. In patients with tuberculosis, it leads to reduction in appetite, nutrient malabsorption, micronutrient malabsorption, and altered metabolism leading to wasting. Both, protein-energy malnutrition and micronutrients deficiencies increase the risk of tuberculosis. It has been found that malnourished tuberculosis patients have delayed recovery and higher mortality rates than well-nourished patients. Nutritional status of patients improves during tuberculosis chemotherapy. High prevalence of human immunodeficiency (HIV) infection in the underdeveloped countries further aggravates the problem of malnutrition and tuberculosis. Effect of malnutrition on childhood tuberculosis and tuberculin skin test are other important considerations. Nutritional supplementation may represent a novel approach for fast recovery in tuberculosis patients. In addition, raising nutritional status of population may prove to be an effective measure to control tuberculosis in underdeveloped areas of world.

## INTRODUCTION

Malnutrition and tuberculosis are both problems of considerable magnitude in most of the underdeveloped regions of the world. It is important to consider, how these two problems tend to interact with each other. The term consumption has been virtually synonymous with tuberculosis throughout the history^1^ and the link between tuberculosis and malnutrition has long been recognized; malnutrition may predispose people to the development of clinical disease and tuberculosis can contribute to malnutrition.^2^ Before the advent of antituberculosis chemotherapy, a diet rich in calories, proteins, fats, minerals, and vitamins was generally considered to be an important, if not essential factor in treatment of tuberculosis. The introduction of specific antituberculosis drugs, however, has so radically altered the management of the disease that the role of diet should be considered in the light of the advances in treatment.

In the 21^st^ century, tuberculosis is still the most frequent underlying cause of wasting worldwide. However, pathophysiology of wasting in tuberculosis remains poorly understood.^3^ The prevalence of widespread malnutrition in the population may be expected to pose some special problems with regard to the control of the tuberculosis in the developing countries from the larger point of view of prevention and therapeutic management of individual cases, from the narrower clinical point of view.

The direct evidence of effect of nutrition on tuberculosis is difficult because of whole complex of coincident environmental factors. Despite these limitations, the weight of evidence still favors the view that malnutrition may be an important factor in the high mortality and morbidity from tuberculosis in population subjected to food shortage.^4^ In any consideration of the role of nutrition in tuberculosis, three important questions arise.^4^ Does malnutrition predispose to tuberculosis infection? Does malnutrition influence or modify the course of established tuberculosis infection? Do nutritional factors influence the response to chemotherapy?

High prevalence of human immunodeficiency (HIV) infection in the underdeveloped countries further aggravates the problem of malnutrition and tuberculosis. HIV infection is an important risk factor for development of tuberculosis and adversely affects the nutritional status of patient.

## EFFECTS OF MALNUTRITION ON TUBERCULOSIS

There are several experimental, clinical, and epidemiological studies demonstrating the effects of malnutrition on tuberculosis.

### Epidemiological studies

It has been pointed out that tuberculosis mortality rates in different economic groups in a community tend to vary inversely with their economic levels. There are also numerous observations pointing to increase in tuberculosis mortality in various countries during times of war and famine.^5^

There are numerous studies dealing with the effects of different diets on tuberculosis patients, and widely divergent and contradictory claims have been made. Difficulty in interpretation of these studies is influence of other factors on infection. Among tuberculin skin test positive U.S. navy recruits, the risk of tuberculosis was nearly four-fold higher among men who were at least 10% underweight at baseline than in men who were at least 10% overweight.^6^ In a study among 1,717,655 Norwegians, >15-years old who were followed for 8–19 years after intake into a radiographic screening program, the relative risk of tuberculosis among persons in the lowest body mass index (BMI) category was more than five-fold higher than the group in the highest BMI category, and it was independent of sex, age, and radiographic findings.^7^

In another study conducted in London, it was found that Hindu Asians had an increased risk of tuberculosis compared with Muslims. Religion had no independent influence after adjustment for vegetarianism (common among Hindu Asians).^8^ There was a trend of increasing risk of tuberculosis with decreasing frequency of meat or fish consumption. Lacto-vegetarians had an 8.5-fold risk compared with daily meat/fish eaters. Decreased immunocompetence associated with a vegetarian diet may result in increased mycobacterial reactivation.

Poor nutrition among patients who have undergone gastrectomy may be a risk factor for the reactivation of tuberculosis. There are other studies indicating that incidence of tuberculosis is unusually high among malnourished people.^9^

Now it is important to consider how malnutrition can increase risk of tuberculosis. The host protective immune mechanism of infection with *Mycobacterium tuberculosis* depends critically on the interaction and cooperation between monocyte-macrophages and T-lymphocytes and their cytokines.^10^ Substantial experimental evidence suggests that malnutrition can lead to secondary immunodeficiency that increases the host's susceptibility to infection. Increased risk of tuberculosis can result from alteration in the individual protective function of, or the interaction between T-lymphocytes and macrophages because of nutritional insult.^11^

The reactivation of latent or previously subclinical tuberculosis infection may be related, in part, to deteriorating nutritional status.^12^ Protein malnutrition has been identified as an important risk factor for the predisposition to intracellular infection leading to death.^13^

### Animal studies

Though there are several studies on experimental animals that have concluded contributory role of malnutrition on tuberculosis, an important snag in the interpretation of experimental data is the question as to how far these data obtained in the experimental animals are really applicable to human subjects. The natural history and evolution of tuberculosis in the animals and human beings need not necessarily be the same, and of course, the nutritional requirements of the animals and human subjects are also different.^5^

It was reported that protein calorie malnutrition markedly enhanced bacterial growth and dissemination in mice resulting in rapidly fatal tuberculosis infection and a markedly elevated bacillary load in the lungs of protein deficient mice.^11^

Study on guinea pigs demonstrated that protein malnutrition impairs the protective interaction between macrophages and T-lymphocytes and/or the acquisition of mycobactericidal and mycobacteriostatic activity by macrophages in the presence of adequate activation signals.^14^ Protein deficiency prevented guinea pigs from generating a population of antigen-specific-immune lymphocytes and/or impaired the proliferation capacity of these cells.^15^

Cytokines play a central role in mediating antimycobacterial immunity. Interleukin-2 (IL-2) is required to initiate and amplify immune responses. IL-2 production was depressed in chronic protein deficient guinea pigs vaccinated with *M bovis* BCG.^16^ Result of a study on guinea pigs demonstrated that protein malnutrition potentiates the *M. tuberculosis* H37Rv-infected macrophages-monocytes to produce higher levels of transforming growth factor-β (TGF-β) which is a likely mediator of immunosuppression and immunopathogenesis in tuberculosis.^14^

In nutshell, it can be said that malnutrition exerts detrimental effects on many aspects of host immune responses against mycobacterial infection. First, dietary deficiency causes thymic atrophy and impairs the generation and maturation of T-lymphocytes in animal models of tuberculosis, resulting in reduced number of immunocompetent T-cells in lymphoid compartments including the blood. Second, deficiency of protein and other nutrients impair T-cell functions, including decreased production of Th_1_CK IL-2 and IFN-γ and depressed tuberculin reaction and PPD-induced lymphoproliferation in guinea pigs and mice infected with virulent *M. tuberculosis*. Third, protein malnutrition impairs sequestration or trapping of reactive T-lymphocytes and loss of tuberculosis resistance following BCG vaccination. Finally, protein malnutrition potentiates *M tuberculosis* H37Rv infected monocytes-macrophages to produce higher level of TGF-β-a cytokine which has been implicated as a likely mediator of immunosuppression and immunopathogenesis in tuberculosis.

There are some studies which do not prove the role of malnutrition in tuberculosis. A four-year follow-up of treated cases of pulmonary tuberculosis in a study at the Madras Tuberculosis Chemotherapy Center showed that about 90% of patients maintained quiescence throughout the four-years period follow-up in spite of the fact that they were living under great stress of socioeconomic conditions including poor diet. Furthermore, the few patients who had a bacteriological relapse were at no special dietary disadvantage in comparison with those who had quiescent disease throughout.^4^

## EFFECTS OF TUBERCULOSIS ON NUTRITIONAL STATUS

Nutritional status is significantly lower in patients with active pulmonary tuberculosis compared with healthy controls in different studies in Indonesia, England, India, and Japan.^17^ Tuberculosis patients have been found to have lower serum albumin concentration than controls.^17^ Tuberculosis is probably associated with more severe malnutrition than other chronic illnessess; in an Indian study, the nutritional status of patients with tuberculosis was worse than that of those with leprosy. A study in Uganda demonstrated that poor nutritional status is common among adults with pulmonary tuberculosis.^18^ In yet another Indian study, tuberculosis patients were respectively 11 and 7 times more likely to have a BMI < 18.5 and mid-arm circumference < 24 cm.^19^

For any infection, there is a complex interaction between the host response and the virulence of the organisms, which modulates the overall metabolic response and the degree and the pattern of tissue loss. In patients with tuberculosis, a reduction in appetite, nutrient malabsorption, micronutrient malabsorption, and altered metabolism leads to wasting.^20^,^21^

In a study, Indian patients with pulmonary tuberculosis were compared with malnourished and normally nourished healthy subjects. Whereas protein synthesis and breakdown in the fasting state were not significantly different between groups, patients with tuberculosis used a larger proportion of proteins from oral feeding for oxidation and hence for energy production than did either control group. Such failure to channel food protein into endogenous protein synthesis has been termed “Anabolic block”. This anabolic block represents one of the mechanisms for wasting in tuberculosis and other inflammatory status.^21^,^22^

Anorexia is also a contributing factor for wasting in tuberculosis. In an unselected U.S. cohort of patients diagnosed with tuberculosis, 45% lost weight and 20% had anorexia.^23^ Increased production of cytokines with lipolytic and proteolytic activity cause increased energy expenditure in tuberculosis.^24^ Leptin may also play an important role in wasting.^25^ In a study, malnutrition has been associated with atypical presentations of tuberculosis.^26^

### Role of micronutrients

Because of diverse metabolic characteristics and functions, micronutrients have presently been accepted as essential for optimum human health. Micronutrients deficiency is considered to be the most frequent cause of secondary immunodeficiency and infection related morbidity including tuberculosis.

### Zinc

Zinc deficiency affects the host defenses in a variety of ways. It results in decreased phagocytosis and leads to a reduced number of circulating T-cells and reduced tuberculin reactivity, at least in animals.^17^ *In vitro* cellular killing by macrophages was found to be reduced during zinc deficiency and rapidly restored after zinc supplementation.^27^

Various studies on patients with tuberculosis had shown significantly lower plasma zinc level than those without tuberculosis, irrespective of their nutritional status. There was significant rise in zinc level at the end of six- months of antituberculosis therapy (ATT). Thus, it may be suggested that plasma zinc status is likely a marker for monitoring the severity of disease and response to therapy.^17^,^28^,^29^ Zinc deficiency in tuberculosis is likely due to redistribution of zinc from plasma to other tissues or reduction of the hepatic production of the zinc carrier protein α2-macroglobulin and to a rise in the production of metallothionein, a protein that transports zinc to the liver.^17^

Reduction in plasma zinc concentration was shown in tuberculosis patients after two months of treatment. This phenomenon may be because during the intensive phase of ATT, the antituberculosis drugs were used to kill the population of replicating bacilli and zinc may play important role in the macrophage contribution to host defenses at the site of infection.^30^ The other possible mechanisms could be the effect of antituberculosis drugs on zinc absorption. Ethambutol was shown in rats to increase not only zinc absorption but also urinary zinc losses, resulting in reduced circulating zinc concentration.^31^

Zinc supplementation of patients with pulmonary tuberculosis and bacterial pneumonia was shown to increase immune function.^32^ In a study, it was found that PPD indurations were larger in children receiving zinc and zinc increases the PPD induration size in children irrespective of nutritional status.^33^

Zinc has essential role in vitamin A metabolism. Studies in humans and animals have shown that zinc deficiency impairs the synthesis of retinal binding proteins and reduces plasma retinal concentration.^34^ Therefore, it appears that zinc supplementation has a beneficial effect on vitamin A metabolism which has important role in tuberculosis. An adequate supply of zinc may also limit free radical membrane damage during inflammation.^35^

### Vitamin A

It has been shown that vitamin A has immunocompetent role in human tuberculosis. Vitamin A was reported to inhibit multiplication of virulent bacilli in cultured human macrophages.^36^ In addition, vitamin A has a vital role in lymphocyte proliferation and in maintaining the function of epithelial tissues.^37^ Vitamin A is essential for normal functioning of T and B lymphocytes, macrophage activity, and generation of antibody response.^38^

A study from Rwanda reported vitamin A deficiency among adults with tuberculosis. Concentration of vitamin A was found to be lower in tuberculosis patients than that in controls in many studies.^39^,^40^ The low concentration of retinal in plasma can be due to number of factors including reduced intake or reduced absorption of fat. Additionally, infection itself can compromise vitamin A status in number of ways.^41^ Vitamin A is excreted in the urine in patients with fever and this has been confirmed in subjects with acute infection including pneumonia.^42^ During the acute phase response, leakage of proalbumin through the vascular endothelium occurs; and production of retinal binding proteins and prealbumin by liver is reduced.^41^ In addition, requirement of vitamin A during infection is raised by its increased rate of excretion and metabolism.^42^

In an Indian study, the low vitamin A levels observed in tuberculosis patients returned to normal at the end of ATT without vitamin A supplementation.^43^ Vitamin A deficiency increases bacterial adherence to respiratory epithelial cells.^44^

In the prechemotherapeutic era, cod liver oil rich in vitamin A and D was used regularly to strengthen host defence.^45^ Supplementation of vitamin A appears to increase survival among chicks infected with *M. tuberculosis* and enhances both T-lymphocyte and antibody responses to *M. tuberculosis*.^46^

### Vitamin D

Vitamin D plays a role in the function of macrophages, key factor in host resistance in tuberculosis. Abnormalities in vitamin D status have been reported in tuberculosis. Genetic variations in the vitamin D receptor were identified as a major determinant of the risk for tuberculosis in Africans.^47^ Vitamin D deficiency itself was shown to be a risk factor for tuberculosis.^48^ Adults with untreated tuberculosis in Indonesia were shown to have significantly lower 25-hydroxy-vitamin D compared with controls.^49^

Studies have yielded inconsistent findings regarding serum or plasma calcium concentration during tuberculosis. A study in Africa has related hypocalcemia to moderate to extensive radiographic disease.^50^

### Vitamin E

In many studies, concentration of vitamin E was found to be significantly lower in tuberculosis patients than healthy controls.^39^,^51^

### Vitamin C

Studies have linked vitamin C deficiency with tuberculosis.^39^,^51^ In Ethiopians, concentrations of antioxidant vitamin C, vitamin E, and vitamin A were significantly lower in tuberculosis patients and high malonaldehyde concentration was associated with clinical severity.^52^

### Selenium

The essential trace element selenium has an important function in maintaining the immune processes and thus may have a critical role in clearance of mycobacteria. Selenium has been found as significant factor in the relative risk for developing mycobacterial diseases in HIV positive patients.^53^

A recent Indian study measured concentrations of circulating antioxidants and markers of oxidative stress in tuberculosis patients. Results showed lower antioxidant potential (lower levels of superoxide dismutase, catalase, glutathione, and ascorbic acid) and enhanced lipid peroxidation products (malonaldehyde) in tuberculosis patients. Antioxidant potential increased with treatment.^54^

### Iron

Anemia is highly prevalent among adults with pulmonary tuberculosis.^17^ In a study conducted in Ghana, 50% adults with pulmonary tuberculosis had significantly lower hemoglobin than healthy matched controls. Iron deficiency may also be a contributing factor.^55^

In a study, concentration of hemoglobin was lower in tuberculosis patients than that in controls^17^ and those of zinc protoporphyrin (ZPP) were significantly higher than in controls. Elevated concentration of ZPP, a measure of free erythrocyte porphyrin, is indicative of iron deficient erythropoiesis.^56^

There are two explanations for the association of low iron status and infection. One is that anemia results from chronic infection and the other is that iron deficiency would increase susceptibility to infection such as tuberculosis.^17^

### Copper

In a recent study, compared with the control group, the concentrations of iron, zinc, and selenium were significantly lower while that of copper and copper/zinc ratio were significantly higher in the serum of tuberculosis patients.^57^ The serum concentration of zinc increased and serum copper concentration and copper/zinc ratio decreased significantly after antituberculosis chemotherapy.

### Polyunsaturated fatty acids

In a study, eicosanoid synthesis was studied in macrophages of guinea pigs fed with different amounts of omega-6 fatty acids and omega-3 fatty acids. It was concluded that supplementing the diet with (n-3-) fatty acids can affect resistance to *M. tuberculosis*, whereas supplementing with (n-6-) fatty acids does not.^58^

### Cholesterol

Hypocholesterolemia is common among tuberculosis patients and is associated with mortality in miliary tuberculosis cases. *In vitro* studies concluded that cholesterol-rich diet accelerated the sterilization rate of sputum culture in pulmonary tuberculosis patients suggesting that cholesterol should be used as a complementary measure in ATT.^59^

## TUBERCULIN TEST AND MALNUTRITION

In advanced malnutrition, false negative tuberculin reactions are very frequent.^5^ In a study, within a month of starting routine radiographic examination of cases of malnutrition, it was observed that 11 cases of advanced malnutrition were showing evidence of tuberculosis, but with negative Montoux reaction. False negative tuberculin reaction is because of immunosuppression in protein malnutrition states. Nutritional rehabilitation of such children renders the tuberculin test positive in these children.^4^

## HUMAN IMMUNODEFICIENCY VIRUS INFECTION

Tuberculosis and Human imunodeficiency virus (HIV) infection are wasting diseases that frequently occur together. Two studies in Haiti and England showed significant difference between arm muscle circumference in adults with tuberculosis and healthy controls.^60^ Mid-upper arm circumference was significantly lower among HIV positive adults with tuberculosis than in HIV negative adults with tuberculosis.^26^ Mean triceps skin fold thickness is also lower in HIV positive adults with tuberculosis than in HIV negative adults with tuberculosis.^60^

One study demonstrated that there appears to be a relationship among BMI, host immune function, and natural history of HIV in adults with tuberculosis.^61^ Overexpression of TNF-α may be the cause of wasting.

Net protein anabolism is impaired in patients with HIV positive adults with tuberculosis infection and this impairment is significantly more than that seen in patients with HIV or tuberculosis infection alone. Mortality is increased in HIV and tuberculosis patients who have significant wasting.

In malnourished tuberculosis patients, combination of enhanced oxidative stress and decreased concentration of several antioxidants may have important pathogenic consequences in HIV infected tuberculosis patients. Oxidative stress has been shown to enhance HIV replication, to induce the production of several inflammatory cytokines and to promote lymphocytic apoptosis^62^,^63^ and T-cell dysfunction; and could therefore contribute to increased viral replication and progression of immunodeficiency in patients dually infected with HIV and tuberculosis.^64^

### Nutritional needs of children with tuberculosis

The rapid growth periods of infancy and childhood can only be maintained if a child's nutrient intake is optimal. Tuberculosis can cause impaired growth and malnutrition. Provision of adequate energy and nutrients for a child with tuberculosis is very important since the child has increased requirements as a result of both growth and tuberculosis.

Various studies have concluded that all children presenting with malnutrition or failure to thrive must be evaluated for possible tuberculosis. Because children have limited stomach capacity and appetite, meeting nutritional requirement in children presents a difficult challenge.^5^

### Drug nutrient interaction

Concomitant feeding and administration of antituberculosis drugs with food has been shown to result in decreased bioavailability of rifampicin and isoniazid.^65^

Ethambutol has been shown in rats to increase not only zinc absorption but also urinary zinc losses, resulting in reduced circulating zinc concentration.^31^

Peripheral neuropathy is a well-known adverse effect of isoniazid. Though this is reported to be rare in patients receiving dosages of isoniazid of the order of 5 mg/kg, it is frequently found in subjects receiving higher doses.^4^ Isoniazid-induced peripheral neuropathy is frequently observed among poor segments of population because of malnutrition.

Finally, adverse reactions of antituberculosis drugs are risk factors for malnutrition, independent of age, gender, education, occupation, and access to food stuffs.^66^

## NUTRITIONAL STATUS AND CLINICAL OUTCOMES

Nutritional status appears to be an important determinant of clinical outcome during tuberculosis. In an Indian study, 163 patients with tuberculosis were treated either in a sanatorium with a well-balanced diet or at home on a markedly poor diet. The overall treatment response was similar in both groups, however, those receiving better nutrition tended to show more rapid clearance of bacteria and radiographic changes in addition to greater weight gain.^67^

Experimental studies on rats have shown that animals on high protein diet recovered from the negative nitrogen balance phase following an infection more rapidly than those on low protein diet.^5^ Clinical studies have shown that prognosis in case of tuberculosis is decidedly more favorable in subjects with positive nitrogen balance than in those with negative nitrogen balance.^5^

Serum albumin and hemoglobin concentrations have been found as strong predictors of survival in adults with pulmonary tuberculosis.^68^ In another study on tuberculosis patients, albumin, cholesterol, cholinesterase, hemoglobin level, and weight were lower in patients who died than in those who survived.^69^ In a study in Malawi among 1181 patients with tuberculosis, the risk factor for early mortality included age >35 years, HIV seropositivity, and a high degree of malnutrition.^70^ Among adults with moderate to severe malnutrition, 10.9% died in the first four weeks of treatment as opposed to a 6.5% death rate in adults who were normal or had mild malnutrition. Finally, wasting in tuberculosis is associated with impaired physical function.

### Changes in nutritional status during chemotherapy

Studies have shown that nutritional indicators such as anthropometric measurements improve during tuberculosis chemotherapy.^67^ A study conducted in Malawi showed that among 1181 adults with tuberculosis, weight significantly increased after four weeks of treatment, especially in HIV group.

### Nutritional treatment of tuberculosis

Nutritional supplementation may help to improve outcome in tuberculosis patients. A study found that nutritional counseling to increase energy intake combined with provision of supplements, when started during the initial phase of tuberculosis treatment, produced a significant increase in body weight, total lean mass, and physical function after siix weeks. A large proportion (46%) of the early weight gain comprised lean tissue, confirming the findings that tuberculosis can mount a protein anabolic response on feeding. In the same study, patients in the nutritional supplementation group continued to show a greater increase in body weight than control subjects during later follow-up. However, the pattern changed toward deposition of predominantly fat mass, whereas in the control group, the weight gain comprised fat and lean tissue in approximately equal proportions.^71^ As described above, the changes in lean mass could be an underestimate of the actual improvement in nutritional status, given that feeding initially leads to a loss of extracellular water that accumulates in malnourished individuals, including those with tuberculosis.^20^ Accelerating the recovery of lean tissue might help to restore physical functions more rapidly. Restoration of physical function might help to shorten the convalescent period and facilitate earlier return to productive work.^71^ Early restoration of nutrition could also lead to immunologic changes that could enhance the clearance of mycobacteria and reduce infectiousness of patient.

Vitamins and minerals can play important role in treatment of tuberculosis. In a trial among 110 new cases of active tuberculosis, subjects received tuberculosis chemotherapy alone, or in addition to injectable thiamin, vitamin B_6_, and vitamin C, or an oral multivitamin supplement.^72^ All groups receiving any vitamin supplementation had significantly better lymphocyte proliferation responses than the group receiving no supplement. Another trial showed that vitamins C and E were effective in improving immune responses to tuberculosis when given as adjuvant to multidrug tuberculosis therapy.^73^ The supplementation with vitamin A and zinc improved the effectiveness of the antituberculosis drugs in the first two months. The improved outcome was indicated by the higher number of patients with sputum negative for bacilli and significantly lower mean lesion area in the lungs.^30^

A retrospective study on nonmiliary tuberculosis patients admitted in ICU with respiratory failure found that early and aggressive attention to improving the patient's nutrition may be as important as effective ATT and mechanical ventilation in salvaging these patients.^68^

Nutritional supplementation may represent a novel approach for fast recovery in tuberculosis patients. In addition, raising nutritional status of population may prove to be an effective measure to control tuberculosis in underdeveloped areas of world.
